# Changes in Choroidal Circulation Hemodynamics Measured Using Laser Speckle Flowgraphy after a Cold Pressor Test in Young Healthy Participants

**DOI:** 10.3390/tomography9020064

**Published:** 2023-04-06

**Authors:** Sakurako Imabayashi, Yuki Hashimoto, Yumi Ishimaru, Rino Umemoto, Miho Chiyozono, Toshitaka Yamanokuchi, Takeshi Yoshitomi

**Affiliations:** 1 Department of Orthoptics, Faculty of Medicine, Fukuoka International University of Health and Welfare, Momochihama 3-6-40, Sawara-ku, Fukuoka 814-0001, Japan; 2 Department of Physical Therapy, Faculty of Medicine, Fukuoka International University of Health and Welfare, Momochihama 3-6-40, Sawara-ku, Fukuoka 814-0001, Japan

**Keywords:** choroidal circulation hemodynamics, laser speckle flowgraphy, mean blur rate

## Abstract

Using laser speckle flowgraphy (LSFG), we investigated the time course of changes in choroidal circulation hemodynamics after a cold pressor test in healthy eyes. This prospective study included the right eye of 19 young healthy participants. The macular mean blur rate (MBR) was measured with LSFG. The MBR, intraocular pressure (IOP), systolic blood pressure (SBP), diastolic blood pressure (DBP), heart rate (HR), mean blood pressure (MBP), and ocular perfusion pressure (OPP) were evaluated at baseline; immediately after the test and 10, 20, and 30 min later. Immediately after the test (0 min), SBP, DBP, MBP, and OPP were significantly elevated compared with those at baseline. The macular MBR significantly increased by +10.3 ± 7.1% immediately after the test. However, there was no change after 10, 20, and 30 min in the above parameter. A significant positive correlation of the macular MBR with the SBP, MBP, and OPP was observed. In young healthy individuals, increased sympathetic activity induced by a cold pressor test increases choroidal hemodynamics in the macula along with an increase in systemic circulatory dynamics, which normalizes after 10 min. Therefore, LSFG may provide a novel approach for assessing sympathetic activity and intrinsic vascular responsiveness in the eye.

## 1. Introduction

Stress caused by cold exposure activates the sympathetic nervous system, resulting in increased blood pressure (BP) and heart rate (HR); these are known as pressor responses, and cold pressor tests are performed to assess sympathetic responses in systemic circulatory dynamics [1,2,3,4,5]. The cold pressor test is a common stress test for evaluating BP and HR responses to cooling and involves cooling hands, feet, or the whole body. This test is used to screen individuals for hypertension, and the cardiovascular responses may be used to predict future hypertension [1,2].

The choroidal blood supply accounts for 85% of the total ocular blood flow [6]. Autoregulation varies among vascular systems, and choroidal vessels are poorly autoregulated [7,8,9]. Moreover, human and animal studies have also shown some degree of autoregulation of choroidal vessels in response to changes in ocular perfusion pressure (OPP) [10,11]. In addition, the regulatory mechanisms of the choroidal circulation compensate better for increases in BP than for increases in intraocular pressure (IOP), and for the same OPP, blood flow is better regulated at lower, rather than at higher IOPs [11]. However, since the choroid is mainly controlled by sympathetic innervation [7,8,9], choroidal autoregulation is also affected by IOP, BP, OPP, and sympathetic input [12]. It has been reported that the choroidal morphology of healthy participants was quantitatively evaluated using enhanced depth imaging optical coherence tomography using the cold pressor test; the results showed a significant decrease in subfoveal choroidal thickness after the test. In addition, changes in Systolic BP (SBP), diastolic BP (DBP), mean BP (MBP), HR, OPP, IOP, and choroidal morphology from pre-test to immediately post-test were reported to be not significantly different by sex [13].

Laser speckle flowgraphy (LSFG) can be used to noninvasively measure choroidal blood flow and has the advantage of providing reproducible data throughout retinochoroidal diseases [14,15,16,17,18]. The mean blur rate (MBR) is a quantitative index of relative blood flow velocity. It has been reported that sex-related differences are not present in choroidal blood flow in healthy subjects using LSFG [19]. Acute central serous chorioretinopathy (CSC) is a common ocular disease caused by increased sympathetic nerve activity and/or a stress-prone personality. A previous study on acute CSC demonstrated an increased macular MBR at the acute stage, validating choroidal blood flow elevation as a factor in the pathogenesis of acute CSC [15,16]. Furthermore, a recent study reported that the macular MBR increases in the acute phase of hypertensive chorioretinopathy, caused by choroidal hyperperfusion [17].

Additionally, previous reports of a significant increase in choroidal blood flow velocity with increasing IOP, BP, and OPP after isometric exercise in healthy individuals indicate that choroidal circulatory dynamics are dependent on systemic circulatory dynamics [9,11,20].

Therefore, it is hypothesized that the choroidal circulations will also increase in conjunction with the increase in systemic circulations by the cold pressor test. This may lead to the prediction and early detection of retinochoroidal diseases and the classification of their severity. However, changes in choroidal blood flow with LSFG after an increase in sympathetic activity induced by a cold pressor test have not yet been assessed. In this study, we investigated the time course of changes in the MBR after a cold pressor test using LSFG.

## 2. Materials and Methods

### 2.1. Participants

This study was approved by the ethics committee of Fukuoka International University of Health and Welfare (approval ID: 20-fiuhw-022) and adhered to the tenets of the Declaration of Helsinki. Written informed consent was obtained from all participants.

This prospective study included the right eyes of 19 young healthy volunteers (4 males, 15 females) with no ophthalmic or cardiovascular diseases (Table S1). All participants were recruited using the voluntary response type of non-probability sampling.

Each participant underwent examinations, including best-corrected visual acuity, fundus photography, IOP, and LSFG. SBP, DBP, and HR were also measured.

### 2.2. Cold Pressor Test

The examinations were carried out in a quiet examination room at room temperature (24 ± 1 °C), and humidity was maintained at 47 ± 3%. During the cold pressor test, the right hand was immersed up to the wrist in ice water at approximately 1 °C for 30 s [5,13]. LSFG, IOP, BP, and HR measurements were assessed at baseline, immediately after the test (0 min), and 10, 20, and 30 min later. At all measurement points, BP and HR were measured first; LSFG was performed second, followed by IOP testing. All examinations were performed in the sitting position, and each experimental session (each measurement time of 90 s for BP and HR, 12 s for LSFG, and 15 s for IOP) was completed within 3 min. Additionally, the participants were asked not to smoke or exercise for at least 2 h before the examinations and were allowed to rest for 10 min in a quiet room.

### 2.3. LSFG Measurement

**Figure 1:**
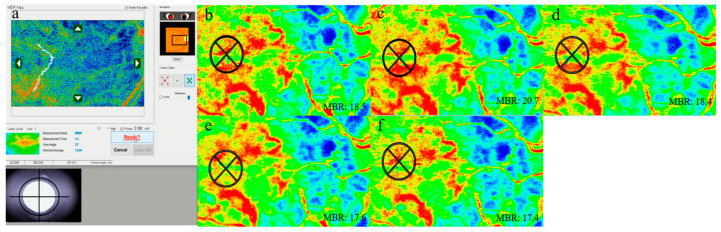
Laser speckle flowgraphy (LSFG) measurement screen (**a**) and the composite color map image of mean blur rate (MBR) at baseline (**b**), immediately after the test (**c**), and at 10 (**d**), 20 (**e**), and 30 (**f**) minutes later in a participant.

LSFG targets moving red blood cells in the deep choroidal vessels using an 830 nm diode laser to illuminate the ocular fundus. The laser speckle method is advantageous because it is quantitative and replicable, and its application to measure ocular blood velocity has been previously reported to yield reproducible results [14,21,22]. LSFG-NAVI (Softcare Ltd., Fukuoka, Japan) was used to measure the hemodynamics of the posterior fundus. The pupils of each participant were dilated with 0.4% tropicamide, 20 min before LSFG testing. Each LSFG examination lasts 4 s, and measurements were obtained in triplicates for each of the evaluation points at baseline, immediately after the test (0 min), and 10, 20, and 30 min later. Large retinal vessels were excluded while evaluating changes in choroidal blood flow velocity at the macula (Figure 1). When participants were followed up, each region of interest (ROI) was automatically set using the LSFG Analyzer software (v 3.0.47; Softcare Ltd., Fukuoka, Japan) at the same site where the ROI was set at baseline. The size of the ROI in the measurement area was standardized for all eyes. To evaluate changes in the average MBR, we used the rate of change of the average MBR against the initial baseline values (defined as 100%) [15,16,17,23].

The measurement screen of LSFG (a). The MBR within the region of interest immediately after the cold pressor test (c) increased by 11.1% compared with that at baseline (b). However, after 10, 20, and 30 min of testing, the results were similar to those at baseline at −1.5% (d), −1.5% (e), and +0.8% (f), respectively.

### 2.4. Hemodynamics and IOP

For all participants, BP and IOP were measured at baseline, immediately after the cold pressor test (0 min), and 10, 20, and 30 min later. The IOP measurement was performed with a noncontact tonometer (NT-530, NIDEK, Gamagori, Japan). Hemodynamics in the eye depend on BP and IOP and are measured using an indicator called OPP, which reflects systemic circulatory dynamics. The OPP was calculated from the IOP and BP values [9]. MBP was calculated from SBP and DBP according to the following equation:

(1) MBP=DBP+1/3(SBP−DBP)

OPP was calculated as:

(2) OPP=2/3MBP−IOP

### 2.5. Statistics

All results are expressed as the mean ± standard deviation. The Friedman test and Scheffe’s paired comparison test were used to examine sequential changes in IOP, SBP, DBP, MBP, HR, OPP, and the MBR. Spearman’s rank correlation test was used to determine the relationships between the rate of change in MBR and other studied factors, including IOP, SBP, DBP, MBP, HR, and OPP. For all tests, *p* values < 0.05 indicated statistical significance.

## 3. Results

The mean age was 22.3 ± 3.3 years (range, 21 to 36 years). All participants had a best-corrected visual acuity score ≥ 20/20.

### 3.1. IOP and Systemic Factors

**Figure 2:**
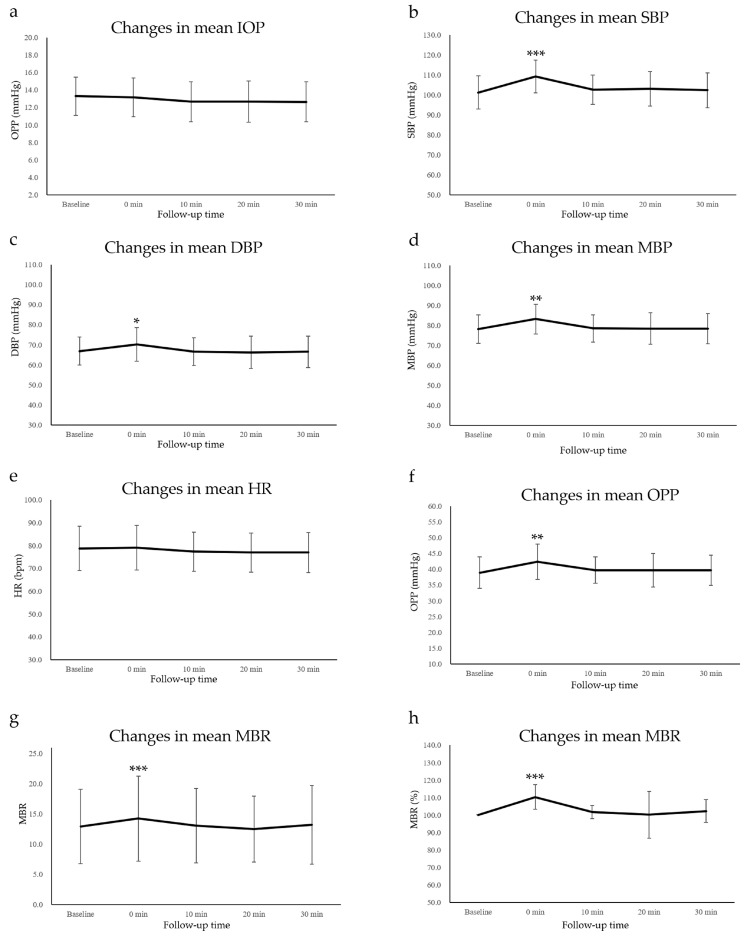
Changes in intraocular pressure (IOP) (**a**), systolic blood pressure (SBP) (**b**), diastolic blood pressure (DBP) (**c**), mean blood pressure (MBP) (**d**), heart rate (HR) (**e**), ocular perfusion pressure (OPP) (**f**), MBR (**g**), and MBR (%) (**h**) at baseline and after the cold pressor tests (* *p* < 0.05, ** *p* < 0.01, *** *p* < 0.001).

Changes in the values of IOP and other systemic factors are summarized in Table 1 and Table S1. The IOP and HR did not change throughout the study (Table 1 and Figure 2a,e). While SBP, DBP, and MBP significantly increased immediately after the cold pressor test (0 min) compared with those at baseline, they showed no changes after 10, 20, and 30 min (Table 1 and Figure 2b–d).

The mean IOP and HR did not change throughout the study (Friedman test = 0.232, 0.565, respectively) (Figure 2a,e). The mean SBP (Figure 2b), DBP (Figure 2c), MBP (Figure 2d), ocular perfusion pressure (OPP) (Figure 2f), MBR, (Figure 2g), and MBR (%) (Figure 2h) significantly increased immediately after the cold pressor test (0 min) compared with those at baseline (*p* < 0.001, *p* = 0.025, *p* = 0.005, *p* = 0.004, *p* < 0.001, *p* < 0.001); they showed no changes after 10, 20, and 30 min.

### 3.2. OPP Data

The changes in the OPP are presented in Table 1 and Table S1. The mean OPP was 38.9 ± 5.0 mmHg at baseline, 42.3 ± 5.6 mmHg at 0 min, 39.7 ± 4.2 mmHg at 10 min, 39.7 ± 5.4 mmHg at 20 min, and 39.7 ± 4.8 mmHg at 30 min after the cold pressor test. The mean OPP value at 0 min was significantly higher than the baseline value; however, there was no significant difference at 10, 20, and 30 min relative to the baseline data (Table 1 and Figure 2f). 

### 3.3. LSFG Data

MBR changes are presented in Table 1 and Table S1. The average macular MBR values at baseline, immediately after the cold pressor test (0 min), and 10, 20, and 30 min later, respectively, were 12.9 ± 6.2, 14.2 ± 7.0, 13.1 ± 6.2, 12.5 ± 5.5, and 13.2 ± 6.5. The macular MBR values significantly increased by +10.3 ± 7.1% immediately after the test, but there was no significant difference at 10, 20, and 30 min relative to the baseline data (Table 1 and Figure 2g,h).

### 3.4. Correlation between MBR and the Other Studied Factors

The correlation between the rate of change in MBR and changes in other studied factors from baseline to a point immediately after the cold pressor test (0 min) was examined. MBR showed a statistically significant positive correlation with SBP, MBP, and OPP (Table 2). However, MBR showed no significant correlation with IOP, DBP, and HR (Table 2).

## 4. Discussion

In this study, SBP, DBP, MBP, OPP, and MBR increased significantly after the cold pressor test but did not vary further after 10 or more minutes. In addition, there was a statistically significant positive correlation of the macular MBR with SBP, MBP, and OPP. These results indicate that an increase in sympathetic activity induced by cold stimulation also increases the choroidal circulation hemodynamics, depending on systemic circulatory hemodynamics, and that these values normalize after 10 min.

Previous reports have shown that both SBP and DBP increase owing to vasoconstriction caused by sympathetic hyperactivity in the cold pressor test [1,2,3,4,5]. Recently, in a report examining the vascular reactivity of the retina and choroid using functional optical coherence tomography, the vascular perfusion density of healthy participants after a cold pressor test decreased in the choroid but remained unchanged in the retina. Furthermore, choroidal vascular perfusion density had a strong, inverse correlation with integrated muscle sympathetic nerve activity [8].

It has been reported that choroidal blood flow velocity increases during the acute phase in sympathetic hyperactivity etiologies such as CSC. There are two possible causes of increased choroidal blood flow in eyes with acute CSC. Firstly, the activation of sympathetic α-adrenergic receptors causes vasoconstriction of the choroidal arteries and secondary overflow into the choroidal vasculature. Secondly, the cardiac output is increased owing to the activation of sympathetic β-adrenergic receptors, which increases blood flow in the choroid [15,16]. Furthermore, the MBR changes in hypertensive chorioretinopathy, where severe hypertension is an etiologic factor, are similar to those observed in CSC [17]. In the present study, choroidal blood flow velocity increased immediately after the cold pressor test, when the activity of the sympathetic nervous system was high, and accordingly, BP and OPP increased. Our LSFG results after the cold pressor test in healthy eyes are comparable to previous observations in CSC and may be described as a “non-inflammatory (sympathetic)” pattern in the choroid [15,16].

Additionally, previous studies showing a significant increase in choroidal blood flow velocity with increasing IOP, BP, and OPP after isometric exercise in healthy individuals indicate that choroidal circulatory dynamics are dependent on systemic circulatory dynamics [9,11,20]. Although IOP, BP, OPP, and neural input need to be considered with regard to choroidal autoregulation [11,12], a particularly pertinent point is that choroidal vessels are predominantly controlled by sympathetic innervation [7,8,9]. The cold pressor test performed in this study can evaluate sympathetic responses in systemic circulatory dynamics [1,2,3,4,5]. Therefore, our data suggest that an increase in sympathetic activity during cold stimulation increases systemic circulatory dynamics and choroidal blood flow velocity.

The primary limitations of this study were as follows: first, the relatively small sample size and the difference in sex ratio and, second, the absence of other choroidal circulatory examinations. To further investigate choroidal changes in healthy participants, the relationship between choroidal function and morphology should be assessed in large and equally sexed samples using both LSFG and optical coherence tomography angiography. Future studies should compare the MBR of CSC eyes and healthy eyes using the cold pressor test to determine the relationship between the severity of CSC and the prediction of future onset.

## 5. Conclusions

In conclusion, to the best of our knowledge, this is the first study to report changes in the MBR after a cold pressor test. Our data suggested that in young healthy individuals, as we hypothesized, an increase in sympathetic activity induced by a cold pressor test increases choroidal hemodynamics in the macula along with an increase in systemic circulatory dynamics, which normalizes after 10 min. These novel findings suggest that LSFG may provide a novel approach for assessing sympathetic activity and intrinsic vascular responsiveness in the eye. In addition, a comparison of the choroidal circulation in CSC eyes and healthy eyes using the cold pressor test may reveal the relationship between the severity of CSC and the prediction of future development.

## Figures and Tables

**Table 1:** Changes in ocular biometric parameters and systemic factors at baseline and after the cold pressor tests.

		Post				Friedman Test (*p* Value)	Scheffe’s Paired Comparison (*p* Value)			
	Baseline	0 min	10 min	20 min	30 min		0 min	10 min	20 min	30 min
IOP (mmHg)	13.3 ± 2.2	13.2 ± 2.2	12.7 ± 2.3	12.7 ± 2.4	12.6 ± 2.3	0.232	0.991	0.783	0.613	0.374
SBP (mmHg)	101.2 ± 8.3	109.3 ± 8.2 ***	102.6 ± 7.3	103.2 ± 8.6	102.4 ± 8.6	<0.001	<0.001	0.443	0.533	0.623
DBP (mmHg)	66.8 ± 7.0	70.2 ± 8.4 *	66.6 ± 7.0	66.2 ± 8.0	66.5 ± 7.9	<0.001	0.025	0.982	0.982	0.996
MBP (mmHg)	78.3 ± 7.1	83.2 ± 7.5 **	78.6 ± 6.8	78.5 ± 7.9	78.4 ± 7.6	<0.001	0.005	0.983	0.994	0.998
HR (bpm)	78.8 ± 9.6	79.1 ± 9.8	77.3 ± 8.5	77.0 ± 8.6	76.9 ± 8.7	0.565	0.999	0.970	0.815	0.951
OPP (mmHg)	38.9 ± 5.0	42.3 ± 5.6 **	39.7 ± 4.2	39.7 ± 5.4	39.7 ± 4.8	0.001	0.004	0.776	0.901	0.931
MBR	12.9 ± 6.2	14.2 ± 7.0 ***	13.1 ± 6.2	12.5 ± 5.5	13.2 ± 6.5	<0.001	<0.001	0.894	0.969	0.987
MBR (%)	100.0 ± 0.0	110.3 ± 7.1 ***	101.6 ± 3.8	100.2 ± 13.4	102.3 ± 6.6	<0.001	<0.001	0.894	0.969	0.987

IOP, intraocular pressure; SBP, systolic blood pressure; DBP, diastolic blood pressure; MBP, mean blood pressure; HR, heart rate; bpm, beats per minute; OPP, ocular perfusion pressure; MBR, mean blur rate; min, minutes. Results are expressed as the mean ± standard deviation. Scheffe’s paired comparison, * p < 0.05, ** *p* < 0.01 *** *p* < 0.001.

**Table 2:** Relationship between MBR and other studied factors.

	Macular MBR	
	Coefficient	*p* Value
IOP	−0.196	0.421
SBP	0.732	<0.001
DBP	0.196	0.420
MBP	0.545	0.015
HR	−0.009	0.704
OPP	0.495	0.030

MBR, mean blur rate; IOP, intraocular pressure; SBP, systolic blood pressure; DBP, diastolic. blood pressure; MBP, mean blood pressure; HR, heart rate; OPP, ocular perfusion pressures.

## Data Availability

All data generated or analyzed during this study are included in this published article and its supplementary information files.

## Supplementary Materials

The following supporting information can be downloaded at: https://www.mdpi.com/article/10.3390/tomography9020064/s1, Table S1: Changes in ocular biometric parameters and Table S2: Changes in systemic factors.
