# Supplementary material for: Changes in Choroidal Circulation Hemodynamics Measured Using Laser Speckle Flowgraphy after a Cold Pressor Test in Young Healthy Participants
Source: Tomography. 2023 Apr 6;9(2):790–7. doi: 10.3390/tomography9020064 (PMC10146766; doi:10.3390/tomography9020064)
Supplement: Supplementary file 1 [file tomography-09-00064-s001.zip › tomography-2178544-supplementary.pdf]

Table S1. Characteristics and changes in ocular biometric parameters at baseline and after the cold pressor test in participants.

Case	Age (years)	Sex	IOP (mmHg)					OPP (mmHg)					MBR					MBR（%）				
			Baseline	0 min	10 min	20 min	30 min	Baseline	0 min	10 min	20 min	30 min	Baseline	0 min	10 min	20 min	30 min	Baseline	0 min	10 min	20 min	30 min
1	21	F	14.0	13.7	14.7	14.7	14.3	43.8	46.3	39.5	43.3	39.5	13.7	14.0	13.3	13.5	13.6	100.0	102.2	97.1	98.5	99.3
2	21	F	13.0	12.7	12.0	11.0	14.0	41.7	44.0	45.8	45.2	41.1	23.4	25.6	22.7	13.6	24.5	100.0	109.4	97.0	58.1	104.7
3	22	F	11.0	9.7	9.7	10.0	10.0	39.7	45.6	41.0	38.2	39.8	10.2	11.1	10.2	10.4	10.2	100.0	108.8	100.0	102.0	100.0
4	21	F	12.0	12.0	11.3	11.0	12.0	40.4	45.6	38.9	43.0	42.0	11.1	11.8	11.2	11.2	11.3	100.0	106.3	100.9	100.9	101.8
5	21	F	13.7	14.3	13.7	14.3	13.0	41.4	44.1	43.2	40.4	40.1	18.0	19.7	19.5	20.0	19.4	100.0	109.4	108.3	111.1	107.8
6	37	M	15.3	15.0	15.0	14.0	14.3	49.1	55.2	50.6	51.1	51.0	11.5	12.7	11.8	11.5	11.4	100.0	110.4	102.6	100.0	99.1
7	22	F	10.7	10.0	8.7	10.0	9.7	37.3	41.6	40.6	38.2	37.9	18.5	19.9	18.5	18.1	17.2	100.0	107.6	100.0	97.8	93.0
8	22	F	11.0	12.0	11.3	11.3	10.3	35.7	38.4	39.4	38.9	38.1	8.4	10.2	9.0	9.9	8.2	100.0	121.4	107.1	117.9	97.6
9	21	F	12.0	11.0	11.3	11.7	12.7	36.2	42.6	36.0	35.0	33.1	7.1	8.2	7.4	7.2	7.1	100.0	115.5	104.2	101.4	100.0
10	21	F	17.3	17.7	15.3	17.3	16.7	37.1	38.3	38.5	33.6	38.9	12.2	13.2	11.6	12.2	12.2	100.0	108.2	95.1	100.0	100.0
11	21	M	13.7	13.0	10.7	11.0	9.3	38.5	43.9	40.9	40.1	42.3	7.8	8.9	8.3	8.0	7.9	100.0	114.1	106.4	102.6	101.3
12	22	M	13.0	12.7	14.3	15.3	15.3	39.4	43.1	39.9	38.7	42.0	7.1	8.5	7.4	8.9	8.8	100.0	119.7	104.2	125.4	123.9
13	21	F	14.0	15.3	15.0	13.3	14.0	34.2	36.9	34.3	34.7	32.0	8.7	10.0	8.6	8.6	8.5	100.0	114.9	98.9	98.9	97.7
14	22	M	11.3	12.7	10.7	10.3	10.7	42.0	41.5	41.1	42.8	42.0	9.9	9.9	10.0	9.8	10.0	100.0	100.0	101.0	99.0	101.0
15	22	F	9.7	10.7	10.7	10.0	10.0	43.9	47.1	39.7	48.4	46.2	5.6	6.4	5.9	6.0	5.8	100.0	114.3	105.4	107.1	103.6
16	22	F	12.0	11.7	12.7	12.3	12.0	44.9	48.7	43.5	43.3	43.8	6.1	6.4	6.3	6.1	6.2	100.0	104.9	103.3	100.0	101.6
17	22	F	18.0	18.0	18.3	17.7	17.3	35.1	30.0	33.7	34.5	35.8	16.8	15.8	16.1	15.2	16.8	100.0	94.0	95.8	90.5	100.0
18	22	F	15.7	14.7	12.0	11.3	11.7	27.0	32.6	31.8	28.3	30.1	24.5	27.2	24.3	20.1	23.9	100.0	111.0	99.2	82.0	97.6
19	21	F	15.0	13.0	13.0	14.3	12.7	31.9	39.0	36.8	36.1	38.0	25.2	31.2	26.3	27.7	28.5	100.0	123.8	104.4	109.9	113.1
Mean	22.6		13.2	13.2	12.7	12.7	12.6	38.9	42.3	39.7	39.7	39.7	12.9	14.2	13.1	12.5	13.2	100.0	110.3	101.6	100.2	102.3
SD	5.7		2.2	2.2	2.3	2.4	2.3	5.0	5.6	4.2	5.4	4.8	6.2	7.0	6.2	5.5	6.5	0.0	7.1	3.8	13.4	6.6

M, male; F, female; R, right; L, left; IOP, intraocular pressure; OPP, ocular perfusion pressure; MBR, mean blur rate; min, minutes; SD, standard deviation

Table S2. Changes in systemic factors at baseline and after the cold pressor test in participants.

Case	SBP (mmHg)					DBP (mmHg)					MBP					HR (bpm)				
	Baseline	0 min	10 min	20 min	30 min	Baseline	0 min	10 min	20 min	30 min	Baseline	0 min	10 min	20 min	30 min	Baseline	0 min	10 min	20 min	30 min
1	104.0	108.0	106.0	109.0	98.0	78.0	81.0	69.0	76.0	72.0	86.7	90.0	81.3	87.0	80.7	89.0	91.0	79.0	88.0	79.0
2	108.0	111.0	110.0	109.0	106.0	69.0	72.0	75.0	72.0	71.0	82.0	85.0	86.7	84.3	82.7	78.0	75.0	77.0	78.0	71.0
3	98.0	103.0	98.0	93.0	94.0	65.0	73.0	65.0	62.0	65.0	76.0	83.0	76.0	72.3	74.7	78.0	79.0	73.0	80.0	83.0
4	104.0	111.0	106.0	105.0	103.0	66.0	74.0	60.0	69.0	70.0	78.7	86.3	75.3	81.0	81.0	64.0	70.0	67.0	69.0	78.0
5	106.0	117.0	110.0	106.0	107.0	71.0	73.0	73.0	70.0	66.0	82.7	87.7	85.3	82.0	79.7	77.0	74.0	76.0	75.0	83.0
6	122.0	134.0	125.0	125.0	124.0	84.0	91.0	85.0	84.0	85.0	96.7	105.3	98.3	97.7	98.0	76.0	81.0	80.0	79.0	81.0
7	90.0	96.0	96.0	91.0	96.0	63.0	68.0	63.0	63.0	59.0	72.0	77.3	74.0	72.3	71.3	91.0	90.0	93.0	86.0	87.0
8	90.0	99.0	100.0	100.0	92.0	60.0	64.0	64.0	63.0	63.0	70.0	75.7	76.0	75.3	72.7	81.0	75.0	79.0	76.0	70.0
9	95.0	115.0	97.0	92.0	94.0	61.0	63.0	58.0	59.0	56.0	72.3	80.3	71.0	70.0	68.7	75.0	78.0	81.0	74.0	76.0
10	103.0	106.0	100.0	99.0	104.0	71.0	73.0	71.0	65.0	73.0	81.7	84.0	80.7	76.3	83.3	64.0	74.0	63.0	67.0	74.0
11	109.0	118.0	102.0	104.0	112.0	63.0	69.0	65.0	63.0	60.0	78.3	85.3	77.3	76.7	77.3	87.0	82.0	82.0	75.0	70.0
12	106.0	115.0	100.0	107.0	106.0	65.0	68.0	72.0	68.0	76.0	78.7	83.7	81.3	81.0	86.0	102.0	107.0	100.0	102.0	102.0
13	93.0	103.0	96.0	96.0	97.0	62.0	66.0	63.0	60.0	55.0	72.3	78.3	74.0	72.0	69.0	78.0	72.0	73.0	73.0	71.0
14	104.0	108.0	107.0	105.0	109.0	68.0	68.0	63.0	67.0	64.0	80.0	81.3	77.7	79.7	79.0	62.0	64.0	69.0	67.0	68.0
15	103.0	110.0	101.0	113.0	105.0	69.0	75.0	63.0	75.0	74.0	80.3	86.7	75.7	87.7	84.3	77.0	80.0	69.0	72.0	69.0
16	104.0	110.0	107.0	104.0	107.0	76.0	81.0	73.0	73.0	72.0	85.3	90.7	84.3	83.3	83.7	77.0	67.0	70.0	65.0	61.0
17	105.0	104.0	102.0	109.0	109.0	67.0	56.0	66.0	63.0	65.0	79.7	72.0	78.0	78.3	79.7	71.0	71.0	76.0	73.0	77.0
18	86.0	103.0	91.0	88.0	84.0	53.0	55.0	53.0	45.0	52.0	64.0	71.0	65.7	59.3	62.7	83.0	87.0	80.0	79.0	84.0
19	93.0	106.0	96.0	105.0	98.0	59.0	64.0	64.0	61.0	65.0	70.3	78.0	74.7	75.7	76.0	87.0	86.0	82.0	85.0	78.0
Mean	101.2	109.3	102.6	103.2	102.4	66.8	70.2	66.6	66.2	66.5	78.3	83.2	78.6	78.5	78.4	78.8	79.1	77.3	77.0	76.9
SD	8.3	8.2	7.3	8.6	8.6	7.0	8.4	7.0	8.0	7.9	7.1	7.5	6.8	7.9	7.6	9.6	9.8	8.5	8.6	8.7

SBP, systolic blood pressure; DBP, diastolic blood pressure; MBP, mean blood pressure; HR, heart rate; bpm, beats per minute; min, minutes; SD, standard deviation
